# Compensatory renal hypertrophy following uninephrectomy is calcineurin-independent

**DOI:** 10.1111/jcmm.12438

**Published:** 2014-10-07

**Authors:** Clintoria R Williams, Brandi M Wynne, Makeeva Walker, Robert S Hoover, Jennifer L Gooch

**Affiliations:** a Atlanta Veterans Administration Medical CenterDecatur, GA, USA; b Department of Medicine/Division of Nephrology, Emory University School of MedicineAtlanta, GA, USA

**Keywords:** renal hypertrophy, cyclosporin, calcineurin, uninephrectomy, NFAT

## Abstract

Calcineurin is a calcium-dependent phosphatase that is involved in many cellular processes including hypertrophy. Inhibition or genetic loss of calcineurin blocks pathological cardiac hypertrophy and diabetic renal hypertrophy. However, calcineurin does not appear to be involved in physiological cardiac hypertrophy induced by exercise. The role of calcineurin in a compensatory, non-pathological model of renal hypertrophy has not been tested. Therefore, in this study, we examined activation of calcineurin and the effect of calcineurin inhibition or knockout on compensatory hypertrophy following uninephrectomy (UNX). UNX induces ∼15% increase in the size of the remaining kidney; the data show no change in the generation of reactive oxygen species (ROS), Nox4 or transforming growth factor-β expression confirming the model as one of compensatory hypertrophy. Next, analyses of the remaining kidney reveal that total calcineurin activity is increased, and, to a lesser extent, transcriptional activity of the calcineurin substrate nuclear factor of activated T cell is up-regulated following UNX. However, inhibition of calcineurin with cyclosporine failed to prevent compensatory renal hypertrophy. Likewise, hypertrophy was comparable to WT in mice lacking either isoform of the catalytic subunit of calcineurin (CnAα−/− or CnAβ−/−). In conclusion, similar to its role in the heart, calcineurin is required for pathological but not compensatory renal hypertrophy. This separation of signalling pathways could therefore help further define key factors necessary for pathological hypertrophy including diabetic nephropathy.

## Introduction

Hypertrophy is a process whereby cells and organs increase in size in response to a stressful stimulus. Hypertrophy can have a positive or negative effect on long-term organ function. For example, the heart undergoes hypertrophy following exercise or in response to chronic hypertension. In the case of hypertension, hypertrophy is a precursor to organ failure while in the setting of exercise hypertrophy is an adaptation that is associated with enhanced function. The differences in the signalling mechanisms that mediate beneficial *versus* pathological hypertrophy are complex and incompletely understood.

Since hypertrophy is often an early indication of disease, there has been a great deal of interest in targeting the mechanisms that drive hypertrophy as a therapeutic strategy. This approach led to the identification of calcineurin as a key mediator of cardiac hypertrophy. Inhibition of calcineurin with cyclosporine A (CsA) blocks hypertrophy induced by aortic constriction, angiotensin II infusion, or constitutive overexpression of hypertrophic-inducing genes [1–3]. Conversely, overexpression of a constitutively active calcineurin transgene in the heart induces cardiac hypertrophy [4]. Evidence for a more complex role for calcineurin in hypertrophy came with the observation that calcineurin inhibition failed to block cardiac hypertrophy in other settings. For example, several groups have now reported that CsA has no effect on exercise-induced hypertrophy [5,6]. One key difference between exercise-induced hypertrophy and pathological hypertrophy is the development of cardiac disease and organ failure which is absent in exercise models and present in pathological models. This led to the hypothesis tested and confirmed by Wilkins *et al*. that calcineurin is selectively involved in pathological cardiac hypertrophy and yet dispensable for compensatory hypertrophy [5].

In the kidney, both pharmacological and genetic approaches have demonstrated that calcineurin is involved in hypertrophy induced by diabetes. CsA and the related calcineurin inhibitor tacrolimus reduce hypertrophy of streptozotocin (STZ)-induced diabetic rodents [7,8]. Moreover, knockout of the β isoform of the catalytic subunit (CnAβ) prevents STZ-induced diabetic renal hypertrophy in mice [9]. Like aortic constriction and hypertension, diabetes is associated with pathological changes and reduced organ function over time. These data support the model that, similar to the heart, calcineurin is required for pathological hypertrophy in the kidney. However, it is not yet known if calcineurin is similarly required for compensatory renal hypertrophy or if it is unnecessary. To answer this question, this study was undertaken to determine the effect of calcineurin inhibition or loss on a model of compensatory renal hypertrophy.

## Materials and methods

### Animal models

CnAα−/− and CnAβ−/− mice were created by Dr. Jon Seidman and Dr. Jeff Molkentin, respectively, and described previously [10,11]. Since the strains have been maintained on mixed genetic backgrounds, all experiments were performed with littermate controls. All mice were maintained in the Atlanta VAMC animal facility and all procedures were first approved by the Atlanta VAMC IACUC. Mice were socially housed in standard shoe box cages with a regular 12 hr light/dark cycle and fed standard rodent chow with the exception of CnAα−/− and littermate control mice which were supplemented with digestive enzyme enriched chow to compensate for a salivary gland defect [12]. Adult wild-type (WT) mice (6–8 weeks of age) were randomly assigned to undergo uninephrectomy (UNX) or a Sham procedure. A subset of WT mice were treated with CsA (20 mg/kg) which was mixed with peanut oil and applied to moistened standard rodent chow daily for 7 days prior to UNX/Sham surgeries and continued for the duration of the study. Likewise, adult CnAα−/− and CnAβ−/− mice and littermate controls were randomly assigned to undergo UNX or Sham. Group sizes ranged from 4 to 7 depending on the availability of littermates. UNX was performed by suturing and removing the right kidney through a small incision in the flank of isoflurane-anesthetized mice. Sham mice underwent the procedure but the right kidney was left in place. All mice were administered buprenorphine at the time of surgery and meloxicam daily for 3 days. At the end of the study, mice were housed overnight in groups of two in metabolic cages and urine was collected for 24 hrs. Renal function was determined by measuring BUN levels using a benchtop Reflotron analyzer and urine creatinine and albumin levels were determined by ELISA (GenWayBio, San Diego, CA, USA).

### Calcineurin activity

Calcineurin activity was determined using a method previously published with minor modifications [13]. Briefly, equal parts lysate, phospho-RII peptide substrate that is synthesized with a TAMRA fluorescent tag, and a reaction buffer (0.1 mg/ml BSA, 35 mM Tris pH 7.5, 25 mM NaCl, 2.0 mM MgCl_2_, 270 μM DTT, 500 μM EDTA, 419 nM okadaic acid [in 0.63% ethanol], 25 mM CaCl_2_) were incubated for 10 min. at 30°. Dephosphorylated peptide was then separated by adding reactions to titanium dioxide-coated 96 well plate followed by gentle shaking for 5 min. The supernatants containing non-bound, dephosphorylated peptide were transferred to a black fluorimetry plate, measured at 485 excitation/528 emission, and units of activity were extrapolated from a standard curve run simultaneously.

### H_2_O_2_ measurement

H_2_O_2_ was measured by horseradish peroxidase-catalyzed oxidation of the non-fluorescent molecule *N*-acetyl-3,7-dihydroxyphenoxazine into the highly fluorescent molecule resorufin (Amplex Red Assay, Invitrogen, Carlsbad, CA, USA). Kidney tissues were sectioned and then incubated in buffer containing 100 μl/ml Amplex Red and 0.2 U/ml horseradish peroxidase for 1 hr at 37°C. Resorufin fluorescence was measured at excitation and emission wavelengths of 540 and 590 nm, respectively. Sample fluorescence was compared with that generated by a H_2_O_2_ standard and normalized to tissue weight.

### qRT-PCR

Total RNA was isolated from kidney tissue with Trizol according to the manufacturer's protocol (Invitrogen). cDNA was amplified using One-Step SYBR Green (Bio-Rad, Hercules, CA, USA). All data were normalized to the GAPDH content of the same sample and mRNA expression was calculated using the ΔΔCt method [14]. Primer sequences have been previously published [15].

### Western blot

Flash frozen kidney tissues were lysed in TNESV (50 mM Tris-HCl pH 7.4, 2 mM EDTA, 1% NP-40, 100 mM NaCl, 100 mM Na orthovanadate, 100 μg/ml leupeptin, 20 μg/ml aprotonin and 10^−7^ M phenylmethylsulfonyl) and were then homogenized by douncing on ice. Tissue samples were centrifuged, lysates collected and 20 μg of total proteins were separated by SDS-PAGE. Nox4 and GAPDH were detected as previously described [15].

### Statistical analyses

All data were analyzed using GraphPad Prism (LaJolla, CA, USA) graphing and statistical software. Student's *T*-test, anova, or two-way anova with Bonferroni's post-test were used as appropriate. Results were considered significant and indicated as such if *P* < 0.05.

## Results and discussion

**Fig. 1:**
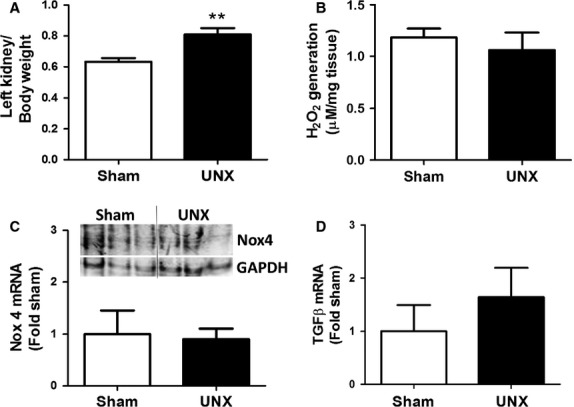
UNX-induced renal hypertrophy is not associated with oxidative stress or fibrosis. (**A**) Left kidney weight was normalized to body weight for UNX and Sham-operated mice. Data shown are the mean ± SEM of five mice per group. *P < 0.05 (Student's *T*-test). (**B**) ROS generation was measured in kidney tissue from UNX and Sham-operated mice by Amplex Red assay and normalized to tissue weight. Data shown are the mean ± SEM of duplicate reactions from five mice per group. (**C**) Expression of the Nox4 isoform of NADPH oxidase was examined by qRT-PCR and western blot in UNX and Sham-operated mice. Data shown are the mean ± SEM of duplicate reactions from five mice per group for qRT-PCR and kidney lysates from three representative mice per group for western blot. GAPDH was also detected as a loading control. (**D**) Expression of TGF-β mRNA was examined by qRT-PCR. Data shown are the mean ± SEM of duplicate reactions from five mice per group.

In humans, UNX is performed for living donation of a kidney. Several decades of follow-up of donors show that, although the remaining kidney quickly undergoes hypertrophy, UNX is associated with very little risk of developing renal disease over the donor's lifetime [16,17]. Similarly, rodents have been followed up to 6 months post-UNX with no signs of renal pathology in the remaining kidney [18]. Uninephrectomy was therefore utilized to assess the role of calcineurin in compensatory hypertrophy. Mice underwent surgical UNX of the right kidney or a Sham procedure using established techniques [19]. Seven days later, left kidneys were harvested for analyses. As expected, UNX induced a statistically significant increase in the size of the remaining kidney (*P* < 0.01) confirming the procedure as a model of hypertrophy (Fig. 1A). One of the key differences between pathological and compensatory hypertrophy is the absence of disease markers including oxidative stress and fibrosis. To confirm that UNX was in fact compensatory rather than pathological, ROS generation by the remaining kidney was examined and compared to Sham treatment. Figure 1B shows that there is no difference in the generation of H_2_O_2_ in kidney samples from UNX mice compared to Sham. Furthermore, the data show that there is no change in mRNA or protein expression of the Nox4 isoform of NADPH which is closely associated with diabetic nephropathy [19,20] (Fig. 1C). Similarly, there is no change in expression of transforming growth factor (TGF)-β, a key pro-fibrotic cytokine that is implicated in nephropathy [22,23], following UNX (Fig. 1D). These findings are consistent with UNX inducing a non-pathological, compensatory hypertrophy.

**Fig. 2:**
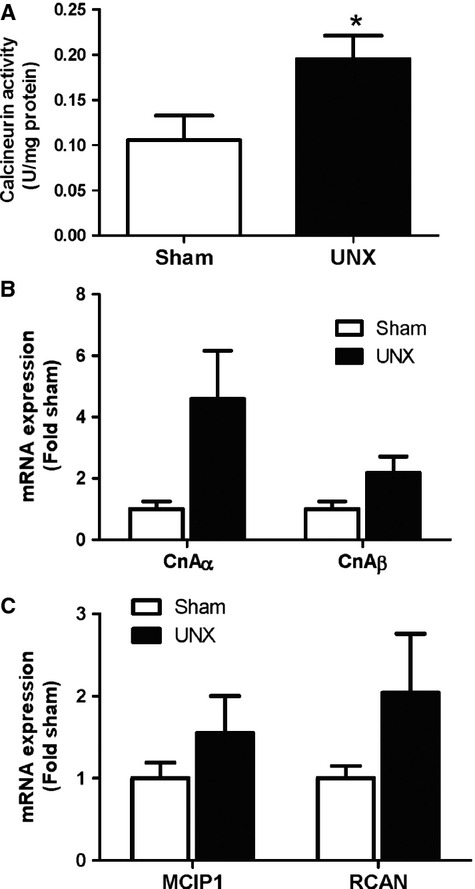
Calcineurin/NFAT activity are increased with UNX. (**A**) Calcineurin activity was measured using an *in vitro* assay. Data shown are the mean ± SEM of duplicate reactions from four to six mice per group. *P < 0.05, Student's *T*-test. (**B**) Expression of CnAα and CnAβ mRNAs were examined by qRT-PCR. Data shown are the mean ± SEM of duplicate reactions from five mice per group. (**C**) Activity of the calcineurin substrate NFAT was assessed by measuring transcription of two target genes – MCIP1 and RCAN – by qRT-PCR. Data shown are the mean ± SEM of duplicate reactions from five mice per group.

To examine the role of calcineurin in compensatory hypertrophy following UNX, calcineurin activity and expression were examined. First, enzyme activity was assessed using an *in vitro* assay. The data indicate that total calcineurin is increased in the remaining kidney following UNX (*P* < 0.05; Fig. 2A). There is also a trend towards an increase in expression of both isoforms of the catalytic subunit (CnAα and CnAβ) although the changes do not reach statistical significance (Fig. 2B). Previously, we showed that nuclear localization of the calcineurin substrate nuclear factor of activated T cells (NFAT) was increased in the diabetic kidney and *in vitro* NFAT is activated by TGF-β [7,24]. Therefore, we examined activity of NFAT following UNX by evaluating mRNA expression of two NFAT-responsive target genes, MCIP1 and RCAN1, by qRT-PCR. Similar to the expression of CnAα and CnAβ, there is a trend toward an increase in NFAT activity with UNX compared to Sham controls although the changes do not reach significance (Fig. 2C). These data suggest that calcineurin/NFAT activity are likely increased with UNX. *In vitro*, calcineurin/NFAT are activated within minutes of stimulation with TGF-β or IGF-I although the pathway is required for long-term changes including hypertrophy and up-regulation of matrix proteins [24,25]. As such, it is possible that maximal calcineurin/NFAT activation occurs earlier than the 7-day post-UNX time-point examined in this study. The changes we observed may have reached statistical significance had we examined kidneys closer to the onset of hypertrophy. Conversely, it is possible that the small changes in calcineurin/NFAT reflect a general increase in expression of proteins involved in basic cellular processes which would accompany compensatory hypertrophy.

To determine if calcineurin is required for compensatory hypertrophy, UNX or a Sham procedure were performed on four experimental groups: WT mice, WT mice treated with 20 mg/kg CsA daily for 1 week prior to surgery and 7 days after, CnAα−/− mice, and CnAβ−/− mice. Table 1 shows that UNX had no significant effect on body weights of the mice but significantly increased blood urea nitrogen (BUN) levels in WT and Cnβ−/− mice. BUN levels of CsA Sham mice and CnAα−/− Sham were elevated compared to WT, consistent with previously published data [26,27] and there was no further increase in BUNs in these groups with UNX. Urine output of all UNX mice was comparable to Sham controls indicating that the remaining kidneys were successfully compensating following UNX (urine output of CnAα−/− mice is increased compared to controls, consistent with a previously published defect in urine concentration [28]). Similarly, there was no increase in urine albumin excretion or urine albumin to creatinine ratio (ACR) with UNX. These data indicate that kidney function was not impaired by UNX in any of the groups.

**Table 1:** Kidney function following UNX

	*N*	Body weight (g)	BUN (mg/dl)	U. output (μl/min)	U. albumin (mg/μl)	U. ACR
WT Sham	7	26.7 ± 1.7	23.3 ± 2.4	0.43 ± .02	3.15 ± 0.66	0.58 ± 0.29
WT UNX	7	24.1 ± 2.2	33.8 ± 1.7**	0.48 ± .01	2.20 ± 0.88	0.56 ± 0.31
CnAα−/− Sham	5	22.9 ± 1.5	**60.0 ± 9.4**##	0.74 ± .01	3.98 ± 1.07	0.38 ± 0.17
CnAα−/− UNX	6	21.6 ± 2.1	64.0 ± 10.9	0.89 ± .01	2.26 ± 0.46	1.35 ± 0.56
CnAβ−/− Sham	4	24.3 ± 1.4	25.8 ± 0.8	0.43 ± .01	3.27 ± 0.57	0.30 ± 0.12
CnAβ−/− UNX	7	23.8 ± 2.4	31.5 ± 1.2*	0.44 ± .01	1.04 ± 0.57	0.86 ± 0.50
CsA Sham	6	19.6 ± 1.3	**30.0 ± 2.5**#	0.57 ± 0.2	1.36 ± 0.39	1.31 ± 0.91
CsA UNX	6	18.1 ± 1.0	41.6 ± 4.6	0.50 ± .08	3.32 ± 1.35	3.90 ± 3.3

** *P* < 0.01 and

* *P* < 0.05 compared to Sham (two-way anova);

## *P* < 0.01 and

# *P* < 0.05 compared to WT Sham (anova).

**Fig. 3:**
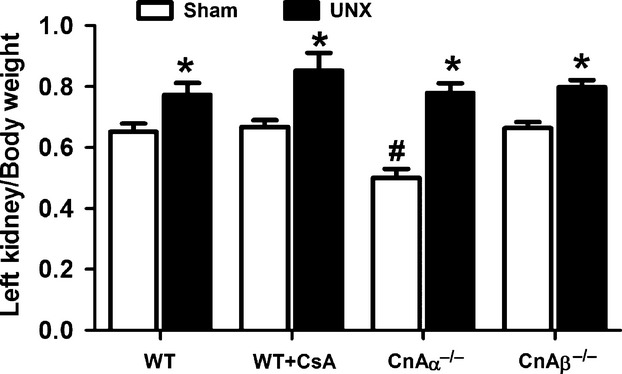
Compensatory hypertrophy following UNX is *calcineurin independent*. Left kidney weight of UNX mice were normalized by body weight and compared with Sham mice for each group. Data shown are the mean ± SEM of 4–7 mice per group. *P < 0.05 compared to Sham (two-way anova); #*P* < 0.05 compared to WT (anova).

Finally, renal hypertrophy was examined in CsA-treated and CnAα−/− and CnAβ−/− mice. Figure 3 shows that, similar to WT, kidneys from UNX CnAα−/− and CnAβ−/− mice are significantly larger than Sham-operated controls within each group (*P* < 0.05, two-way anova). The kidney mass of CnAα−/− Sham mice are smaller than WT as previously reported (*P* < 0.05, anova) [20,21,27,29]. In addition, inhibition of both isoforms by administration of CsA also failed to block hypertrophy. These data indicate that calcineurin is not required for compensatory hypertrophy and that knockout of either isoform as well as inhibition of both with CsA does not impair recovery of urine output or produce any additional pathology [26,27]. As such it is likely that the increase in calcineurin/NFAT activity and expression shown in Figure 2 is a consequence of hypertrophy rather than a requirement for hypertrophy.

In summary, these data indicate that calcineurin is not required for compensatory renal hypertrophy. These findings are significant because they add to the understanding of calcineurin as a selective mediator of pathological hypertrophy, a role that may warrant additional focus as a potential therapeutic target. It is notable that in both the heart and kidney, knockout of the CnAβ isoform is sufficient to attenuate pathological hypertrophy [9,10]. *In vitro*, CnAβ is also sufficient to regulate cellular hypertrophy of renal fibroblasts in response to high glucose and, *via* NFAT, plays a role in up-regulation of Nox 4 [15]. Future studies will likely focus on this pathway as a potential site of therapeutic intervention. In addition, the data clearly establish that there are distinct mechanisms that drive pathological *versus* compensatory hypertrophy in the kidney. Similar to calcineurin, Nox4 is involved in diabetic nephropathy [20,21,30] but its expression and activity are not up-regulated with compensatory renal hypertrophy. This study, however, does not identify other factors which may be required, a limitation that should be addressed in the future.

Finally, it is intriguing that renal hypertrophy can be achieved in a manner that does not lead to pathology. UNX stimulates an increase in renal size and function as demonstrated by normal urine output levels within 1 week of UNX. Unlike diabetes, this was not accompanied by an increase in ROS generation or TGF-β expression. Literature from other investigators confirms that hyperfiltration without proteinuria or hypertension is evident as long as 52 weeks after UNX [18] suggesting that the 7-day end-point in this study did not underestimate these changes. Further work to understand the mediators of compensatory hypertrophy may therefore also be beneficial to the development of novel therapeutic strategies.
